# Burnout and associated factors among nurses working in public hospitals of Harari region and Dire Dawa administration, eastern Ethiopia. A cross sectional study

**DOI:** 10.1371/journal.pone.0258224

**Published:** 2021-10-29

**Authors:** Deribe Bekele Dechasa, Teshager Worku, Negga Baraki, Bedasa Taye Merga, Henock Asfaw

**Affiliations:** 1 School of nursing and midwifery, College of Health and Medical Science, Haramaya University, Dire Dawa, Ethiopia; 2 Department of Environmental Health, College of Health and Medical Science, Haramaya University, Dire Dawa, Ethiopia; 3 School of Public Health, College of Health and Medical Science, Haramaya University, Harar, Ethiopia; Qazvin University of Medical Sciences, ISLAMIC REPUBLIC OF IRAN

## Abstract

**Background:**

Burnout is a syndrome of emotional exhaustion, low personal accomplishment and depersonalization experienced by a health professional and it is more common in nurses due to high workload and job stress that is mostly caused by working proximity to patients and taking care of them. Burnout compromises the provision of quality health care. Despite this, there is no information in Ethiopia on burnout among nurses in study area.

**Objectives:**

To determine the magnitude of burnout and associated factors among nurses working in public hospitals of Harari regional state and Dire Dawa administration, eastern Ethiopia, February 1–29, 2020.

**Methods:**

Institutional based quantitative cross-sectional study was employed from February 1–29 among 412 randomly selected nurses who have been working in hospitals for the last 6 months. Simple random sampling method was employed and data was collected by self-administered, standardized, reliable and valid, questionnaire (Maslachs Burnout Inventory- Human Services Survey). Data was entered into EpiData Version 3.1 and exported to statistical package for social science version 20 for analysis. All covariate with P-value less than 0.25 in bivariable analysis were candidate for multivariable analysis. Level of statistical significance was declared at p-value < 0.05.

**Results:**

Among 412 nurses taking part in this study, 183(44.4%) of nurses with 95% CI, had experienced burnout. Married marital status [AOR:2.3,95%CI:(1.2–4.3)], poor current health status [AOR:4.8, 95% CI:(1.1–21.4)] and fair current health status [AOR:12, 95% CI:(4.5–32)], working greater than eight hour per-day[AOR:0.52, 95%CI:(0.29–0.92)], intention to leave a job [AOR:0.48,95%CI:(0.2–0.88), being working in emergency room [AOR:0.3,95%CI:(0.1–0.98)] and using a different medication related to work related health problems were factors associated with nurses’ burnout.

**Conclusion:**

The nurses’ burnout in this study is high and it is attributed by marriage, perceiving health status as poor and fair, whereas, having the intention to leave job, being working in emergency room and using a medication in relation to work related health problems reduced risk of developing burnout. So, the concerned bodies should provide trainings which focus on stress copying mechanisms and assertiveness program.

## Introduction

The term burnout is first described by Herbert Freudenberg as a condition which was characterized by feelings of emotional exhaustion, disappointment and withdrawal which he initially noticed among voluntary health workers [1]. It has three components, those are: emotional exhaustion, which is feelings of fatigue and of being drained by one’s work; depersonalization, which is the negative attitude towards and a dehumanizing treatment of one’s clients in the work place; and reduced personal accomplishment which has to do with lack of feelings of competence and achievements in one’s work with people [2].

Burnout present in all occupation but health professionals (particularly nurses) are more prone to develop burnout due to the characteristics of their work and due to they spent most of their working hour with clients/patients [3]. The issue of burnout among nurses is more common than other health professionals because nursing is one of the inevitably a stressful profession. Several studies conducted in different continents of the world shows high rates of burnout among nurses, more especially staff nurses working in hospitals and this has a threat to the health care system [4].

The contributing factors to the development of burnout syndrome are: age, gender and marital status [5]. Also mental and emotional factors such as anxiety, stress/depression are also related with burnout syndrome [6]. In addition to these, low salary, shift work responsibility and work load are also associated with burnout [7].

The consequences of burnout are potentially very serious for the nurses, the clients, and the larger institutions in which they interact. It can lead to a deterioration in the quality of care or service that is provided by the nurses. It appears to be a factor in job turnover, absenteeism, low morale, insomnia, increased use of alcohol and drugs, and marital and family problems [8, 9]. Burnout syndrome needs an emphasis since it decrease nurses’ ability to cope up with work related emotional, physical and team work issues [9].

A variety of studies done across the world revealed that the magnitude of burnout among nurses was USA 54.1% [10], Spain 39.8% [11]

Higher burnout were reported among nurse in Africa, In Nigeria the study conducted on nurses indicates a high level of burnout as 39.1% of the respondents in the area of emotional exhaustion (EE), 29.2% in the area of depersonalization and 40.0% in the area of reduced personal accomplishment [12]. Also the study which was done in the same area on nurses but in tertiary hospital of Nigeria shows high level of burnout (42.9%) in area of EE, 47.6% in area of DP and 53.8% in area of reduced personal accomplishment than before [13]. A large scale cross sectional study conducted during the era of COVID-19 among 2014 frontline nurses showed that eight hundred and thirty-five (41.5%) nurses reported high emotional exhaustion, 556 (27.6%) nurses indicated high depersonalization, and 771 (38.3%) had no or low personal achievements, which all indicated high burnout during work [14].

The study which was conducted on health professional working at Jimma University Teaching Hospital (JUTH), to assess the status of burnout among them shows that, from 334 of health professionals, the magnitude of burnout among nurses was 82.75% which was higher than the others’ health professional [15] and also the study which was conducted among nurses working in public hospitals of Amhara regional state reveal high prevalence of burnout among nurses (50.4%) [16].

Burnout among nurses becomes abundant due to multiple complimentary factors such as, work overload, educational status, intention to leave job, health status, mental distress, year of experience, lack of autonomy or authority to make decisions, night and rotating shift in certain cases, inadequate staffing, inadequate resource, anxiety and fear of COVID-19 lead job dissatisfaction, stress and depression which could lead to burnout of healthcare providers and care of the terminally ill patients [17].

Even though many studies were conducted in United State (US), Europe, some parts of Australia and Africa but there is a few studies done in Ethiopia and there is no study conducted on eastern Ethiopian nurses in Harari region and Dire Dawa administration. Hence, this study is aimed to assess nurses’ burnout and its associated factors in public hospitals.

## Materials and methods

### Study area and period

The study was conducted in Harari regional state and Dire Dawa administration. Harari regional state is one of the nine state in Ethiopia and it has a total population of 246,000 of those 124000 are males and the remaining are females [18, 19]. There are one federal police, two public and two private hospitals, eight health centers (four urban and four rural), 19 health posts, 10 non-profit clinics in the Harari region. Two of the public hospitals are Hiwot fana specialized university hospital and Jugal hospital. Hiwot fana specialized university hospital is a teaching hospital of Haramaya University and it has 235 beds and it has 410 health professionals; from this 225 are nurse professionals from which 124 are female nurses and Jugal Hospital is a regional referral hospital with 97 beds and 266 health professional from which nurses are 103. (HFSUH and JH human resource, 2020).

Dire Dawa is located to the eastern part of the Ethiopia. According to the 2007 population and housing census, the population of this city is 342,827 of which more than 67.5% live in urban. It has two public hospitals, the two hospitals are Dilchora referral hospital and Sabian primary hospital [18].

The study was conducted in four public hospitals, two from Harari (HFSUH and JH) and two from Dire Dawa administration (Dilchora referral Hospital and Sabian primary hospital). In all of the four hospitals there are 569 nurses; HFSUH has 225 nurses, JH has 103 nurses, DRH has 163 nurses and SPH has 78 nurses. The data was collected from February 1–29, 2020.

### Study design and population

Institutional based quantitative cross sectional study design was employed. All nurses working in selected public hospitals for last 6 months were included and nurses on sick leave, annual leave, and who are non-volunteer were excluded.

### Sample size determination and sampling procedure

To determine the sample size for this study; outcome variable and the factors that were significantly associated with the outcome variable was considered. The sample size for first and second objective was calculated separately and adding 10% on both specific objectives and the one with the largest number was used for this study.

Single population proportion was used to determine the sample size of the study. By considering the assumption: Z = standard normal distribution (Z = 1.96), with confidence interval of 95%, P = magnitude of burnout among nurses done in Amhara regional state 50.4% [16], d = margin of error = 0.05. Then adding 10% (384×0.1 = 38) of respondents, the total sample was 422.

**Fig 1:**
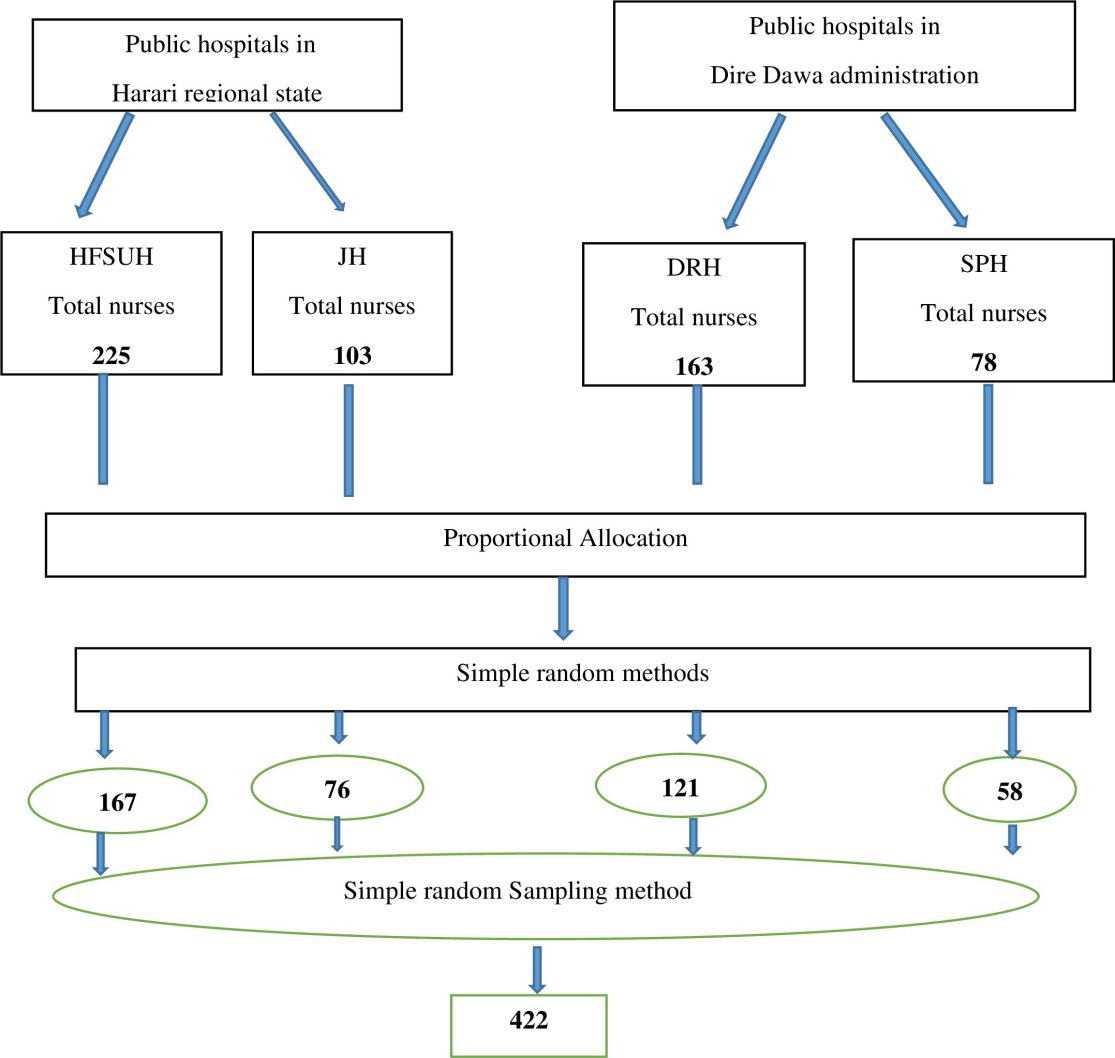
The schematic presentation of the sampling procedure for the study on the magnitude of burnout and associated factors among nurses working in Harari regional state and Dire Dawa city administration, 2020.

Concerning sampling procedure, two hospitals from Harari regional state and two hospitals from Dire Dawa administration are identified for this study. Since there are only four public hospitals in this area, four of them were included in the study and the determined sample size was proportionately allocated to the public hospitals based on the total number of nurses of each public hospitals. The total numbers of nurses in four public hospitals were 569. The sampling frame of nurses were prepared after list of all nurses were taken from each hospitals’ human resource. Nurses were selected from each hospitals and total sample size was met by simple random sampling method based on proportional allocation done for it (Fig 1).

### Data collection tool and procedure

The data was collected by standardized, valid and reliable questionnaire which was adapted from Maslachs Burnout Inventory- Human Services Survey (MBI-HSS [20]. It has three dimension (Emotional Exhaustion (EE), Depersonalization (DP) and Personal achievement (PA)). It was reported that reliability (as measured by Cronbach’s alpha) was 0.9 for EE, 0.79 for DP and 0.71 for PA scale [21]. It has four sections; the first section consists of items for personal information or socio demographic components, the second section consists of items for work related factors, the third section consists of items for organizational and managerial factors and the fourth and the last section comprises 22 items of Maslachs Burnout Inventory- Human Services Survey which was firs advanced by Maslach and Jackson to measure burnout of human service workers.

The total scores of each dimension are summed up and categorized into low, moderate, or high. The cut points of each dimension were; for Emotional exhaustion: low (≤16), moderate (17–26), high (≥27). Depersonalization: low (≤6), moderate (7–12), high (≥13) Personal accomplishment: low (≤31) moderate (32–38), high (≥39) [2]. A nurse is considered to be in a burnout when exhibiting high levels of emotional exhaustion and depersonalization and low regarding personal accomplishment and the aggregation of the rest category of burnouts’ dimensions were represented as no burnout [2].

### Data processing and analysis

The data was checked and entered to Epi-Data version 3.1 and exported to SPSS (Statistical Package for Social Science) version 20 for analysis. The Socio-demographic characteristics and other factors were analyzed by descriptive statistics. All covariate were considered for bivariable analysis and a covariate which has P-value less than 0.25 were analyzed in multivariable analysis. Crude odd ratio and adjusted odd ratio were calculated with the 95% confidence interval to measure the strength of the association between the outcome and independent variables and the variable with P-value less than 0.05 in multivariable analysis was considered as significantly associated with the outcome variable. The logistic regression model fitness was checked using Hosmer and Lemeshow and statistical not significant was declared as model fitted.

### Data quality control

The questionnaire that was developed in English language was translated to Amharic language and then translated back to English language to see the consistency. Brief orientation was given for the study participants. Pre-test was conducted before data collection on 5% of the final sample size in Haramaya hospital which is found in Haramaya town east Hararghe zone. The principal investigator was monitored the whole data collection process and checked for the completeness of data. Intensive supervision was made by supervisors and principal investigator and data double entry was done to make comparisons of two data clerks and resolved if there is some difference.

### Ethical consideration

An ethical clearance was obtained from Institutional Health Research Ethics Review Committee (IHRERC) of Haramaya University College of health and medical sciences and support letter was written to all the 4 public hospitals in which the study was conducted. Informed, voluntary, written and signed consent was obtained from each participant and heads of the respective hospitals. The study had no any risk other than taking a few minutes in filling the data. The information that the participant gave was kept confidentially and there is no any information that identify the participant specifically. The finding of the study was general for the whole nurse staff and wasn’t reflect the particular participant particularly. The participant had the right to withdraw from filling the questionnaire and also has the right to participate in the study.

## Results

### Socio-demographic characteristics

A total of 412 nurses had participated in this study with a response rate of 97.6%. The analysis of the socio-demographic factors showed that 222(53.9%) were females and 190(46.1%) were males and almost half 198(48.1%) of them were between the age of 20–29. The median age of the study participants was 30 ± 8. More than half; 241(58.5%) of the study participants were married. Majority, 297(72.1%) of the study participants were B.Sc. holders and 103(25%) were diploma nurses. Concerning work experience, around one third 135(32.8%) of the study participants had less than 3 years of experience and more than half, 222(53.9%) of the study participants had a monthly income greater than equal to 5295 Ethiopian Birr. Most; 359(87.1%) of the study participants were staff nurses who were carrying out normal nursing routine activity. About 228(53.3%) of the study participants had perceive their current health status as fair, 204(49.5%) and 146(35.4%) of study participants had experienced backache and headache respectively. More than half; 226(54.9%) of the study participants were using analgesic medication (Table 1).

**Table 1:** Sociodemographic characteristics of nurses who were working in public hospitals of Harari regional state and Dire Dawa administration, 2020 (n = 412).

Variables	Respondent	Frequency	Percentage (%)
**Sex**	Male	190	46.1
	Female	222	53.9
**Age**	20–29	198	48.1
	30–39	161	39.1
	40–49	50	12.1
	≥50	3	0.7
**Religion**	Muslims	138	33.3
	Orthodox	173	42.0
	Protestant	84	20.4
	Catholics	12	2.9
	Others*	6	1.5
**Marital status**	Single	164	39.8
	Married	241	58.5
	Separated	7	1.7
**Educational level**	Diploma	103	25
	B.Sc.	297	72.1
	M.Sc.	12	2.9
**Job experience in year**	≤3	135	32.8
	4–6	102	24.8
	7–9	58	14.1
	10–12	46	11.2
	13–15	24	5.8
	≥16	47	11.4
**Monthly income in birr**	≤3653	76	18.4
	3654–4446	45	10.9
	4447–5294	69	16.7
	≥5295	222	53.9
**Job position**	Staff	359	87.1
	Head	33	8.0
	Specialist	13	3.2
	Others**	7	1.7
**Health perception**	Poor	45	10.9
	Fair	228	55.3
	Good	139	33.7
**Health problems**	Headache	146	35.4
	Backache	204	49.5
	Insomnia	27	6.6
	Hypertension	18	4.4
	Others***	17	4.1
**Medication used**	Anxiolytics/sleeping pill	21	5.1
	Analgesic	226	54.9
	Smoking	21	5.1
	Physical activity	125	30.3
	Others****	19	4.6

Others* = Wakefata.

Others** = free servant.

Others*** = Antihypertensive.

### Work/job characteristics of the nurses

The analysis of work related factors showed that 183(44.4%) of nurses were working >8 hour and 229(55.6%) of them were working ≤ 8 hour. Regarding to the working shift, 148(35.9%) were on the day duty and 147(35.7%) were on the night duty and the rests were working alternatively and almost half, 204 (49.5%) of the nurses were care 6–10 patients per day and 78 (18.9%) of nurses care >11 patients per day. From the nurses who participated in the study, 299 (72.6%) of the nurses had no intention to leave their job (Table 2).

**Table 2:** Work/job characteristics of nurses who were working in public hospitals of Harari regional state and Dire Dawa administration, Ethiopia, 2020 (n = 412).

Variables	Respondents	Frequency	Percentage (%)
**Working >8 hour**	Yes	183	44.4
	No	229	55.6
**Working shift**	Day	148	35.9
	Night	147	35.7
	Alternative	117	28.4
**Number patient cared per-day**	≤5	129	31.3
	6–10	204	49.5
	≥11	78	18.9
**Intention to leave job**	Yes	112	272
	No	299	72.6
**Working unit**	Medical ward	75	18.2
	Surgical ward	120	29.1
	Pediatric ward	66	16.0
	Emergency room	77	18.7
	Intensive care unit	40	9.7
	Gynecology/obs	34	8.3

### Organizational and managerial factors

The analysis of organizational and managerial factors indicated that 351(85.2%) of the nurses think that, the hospital has no adequate resource. Regarding their perception on their work satisfaction, more than half, 232(56.3%) of the study participants were perceive their work satisfaction as fair. About 225 (54.6%) of the study participants did not agrees on the presence of the clear communication between the nurses and management. Almost half, 203(50.7%) of the nurses weren’t experienced a conflict with nurses while 201 (48.8%) experienced a conflict with doctors. More than half; 215(52.2%) of the nurses perceived their quality of life as fair (Table 3).

**Table 3:** Characteristics of organizational and managerial factors of Harari regional state and Dire Dawa administration, Ethiopia, 2020 (n = 412).

Variables	Respondents	Frequency	Percentage %
**Resource adequacy**	Yes	61	14.8
	No	351	85.2
**Work satisfaction**	Poor	96	23.3
	Fair	232	56.3
	Good	84	20.4
**Clear communication between nurses**	Yes	187	45.4
	No	225	54.6
**Conflict with nurses**	Yes	203	49.3
	No	209	50.7
**Conflict with doctors**	Yes	201	48.8
	No	211	52.2
**Perception of QOL**	Poor	87	21.1
	Fair	215	52.2
	Good	110	26.7

### Magnitude of burnout among nurses

**Fig 2:**
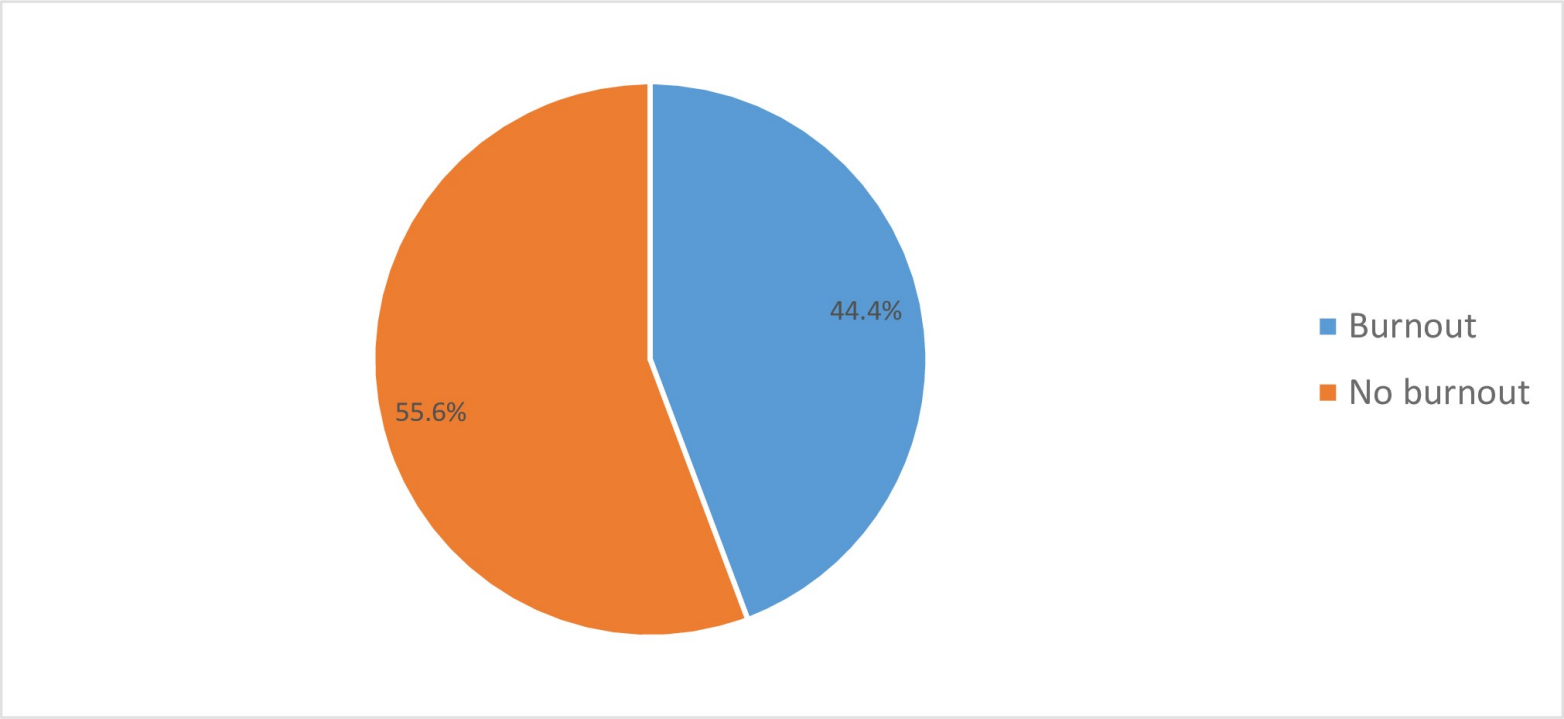
Magnitude of burnout among nurses who were working in Harari regional state and Dire Dawa administration, Ethiopia, 2020.

Among 412 nurses participated in the study, 183(44.4%) of the nurses experienced burnout syndrome. From those 269(65.3%) were high emotional exhaustion, 291(70.6%) were high depersonalization and 307(74.5%) were low personal achievements. A nurse is said to be experienced burnout if she/he scored high in both emotional exhaustion and depersonalization but low score in personal achievements (Fig 2).

### Factors associated with nurses’ burnout

Bivariable binary logistic regression analysis was done and among those variable; sex, age, marital status, educational level, monthly income, job experience, current health status, health problem, medication used, having the intention to leave a job, working unit, hospital resource, perception on work satisfaction, communication between nurse and management and perception of quality of life had association with the nurses’ burnout.

All the variables which have an association with burnout with a P-value less than 0.25 have passed for multivariable logistic regression model analysis. After multivariable logistic regression analysis was done, married nurses, having the intention to leave the profession, perceiving health status as fair and poor, using a medication and working unit have showed the association with burnout among nurses.

Married nurses were 2.3(AOR = 2.269, 95% CI: 1.2–4.3) time more likely at risk for burnout than nurses who were single and nurses who had a perception of their current health status as poor were 5.0(AOR = 5.0, 95%CI: 1.12–22.5) times more likely at risk for burnout than nurses who had a good perception regarding their health status. Additionally nurses who perceive their health status as fair were 13.7 times (AOR = 13.7, 95% CI: 5.24–35.9) more likely at risk for burnout than nurses who had a good perception regarding their health status.

Nurses who had a plan to leave their profession within 12 months were 0.48(AOR = 0.479, 95% CI: 0.26–0.88) less likely at risk for burnout than nurses who had no intention to leave their profession. Nurses who used a medication due to work related health problems were less likely to experience burnout (Table 4).

**Table 4:** Factors associated with burnout among nurses who were working in Harari regional State and Dire Dawa administration, Ethiopia, 2020(n = 412).

Variables	Nurses’ burnout				
	Yes	No	COR(95%CI)	P-value	AOR(95%CI)
**Sex**	76(41.5%)	114(49.8%)	0.72(0.48–1.16)	0.50	0.84(0.50–1.40)
**Male**					
**Female**	107(58.5%)	115(50.2%)	1.00		
**Total**	183(100%)	229(100%)			
**Age**					
**20–29**	80(43.72%)	118(51.53%)	1.00		
**30–39**	76(41.5%)	85(37.1%)	1.32(0.86–2.01)	0.40	0.71(0.32–1.56)
**40–49**	26(14.22%)	24(10.5%)	1.6(0.86–2.98)	0.71	1.35(0.28–6.60)
**> = 50**	1(0.55%)	2(0.87%)	0.74(0.07–8.3)	0.54	0.36(0.01–9.10)
**Total**	183(100%)	229(100%)			
**Marital status**					
**Single**	56(30.6%)	108(47.2%)	1.00		
**Married**	124(67.8%)	117(51.1%)	2.04(1.34–3.1)	0.012	2.3(1.2–4.3)*
**Separated**	3(1.64%)	4(1.75%)	1.45(0.3–6.7)	0.52	1.96(0.25–15.40)
**Total**	183(100%)	229(100%)			
**Educational level**					
**Diploma**	117(63.9%)	84(36.7%)	4.2(1.1–15.9)	0.37	2.18(0.39–12.17)
**B.Sc.**	63(34.4%)	136(59.4%)	1.4(0.4–5.31)	0.84	0.83(0.15–4.63)
**M.Sc.**	3(1.64%)	9(3.93%)	1.00		
**Total**	183(100%)	229(100%)			
**Job experience**					
≤**3**	48(26.2%)	87(38%)	1.00		
**4–6**	45(24.6%)	57(24.9%)	1.4(0.85–2.4)	0.25	1.6(0.72–3.50)
**7–9**	32(17.5%)	26(11.4%)	2.2(1.2–4.2)	0.57	1.34(0.49–3.68)
**10–12**	20(10.9%)	26(11.4%)	1.4(0.71–2.76)	0.91	1.07(0.34–3.45)
**13–15**	16(8.7%)	8(3.5%)	3.6(1.45–9.1)	0.47	1.65(0.42–6.49)
≥**16**	22(12%)	25(10.9%)	1.6(0.81–3.13)	0.46	0.52(0.09–2.95)
**Total**	183(100%)	229(100%)			
**Current health**					
**Poor**	5(2.73%)	18(7.86%)	3.12(0.93–10.77)	0.04	5.0(1.12–22.5)*
**Fair**	170(92.9%)	120(52.4%)	16.12(7.5–34)	0.000	13.7(5.24–35.9)***
**Good**	8(4.4%)	91(39.74%)	1.00		
**Total**	183(100%)	229(100%)			
**Health problems**					
**Headache**	52(28.4%)	94(41.1%)	0.23(0.08–0.69)	0.53	0.63(0.15–2.68)
**Backache**	101(55.2%)	103(44.98%)	0.4(0.14–1.2)	0.79	0.83(0.21–3.32)
**Insomnia**	13(7.1%)	14(6.11%)	0.39(0.11–1.4)	0.60	0.64(0.12–3.50)
**Hypertension**	5(2.7%)	13(5.7%)	0.16(0.04–0.7)	0.78	1.33(0.18–10.07)
**Others**	12(6.6%)	5(2.2%)	1.00		
**Total**	183(100%)	229(100%)			
**Medication used**					
**Anxiolytics/sleeping pill**	5(2.7%)	16(7.00%)	0.02(0.002–0.17)	0.007	0.03(0.002–0.38)**
**Analgesic**	90(49.2%)	136(59.4%)	0.04(0.005–0.28)	0.006	0.04(0.004–0.39)**
**Smoking**	8(4.4%)	13(5.7%)	0.034(0.004–0.31)	0.008	0.034(0.003–0.41)**
**Physical activity**	62(33.9%)	63(27.5%)	0.055(0.007–0.422)	0.006	0.042(0.004–0.412)**
**Others**	18(9.84%)	1(0.44%)	1.00		
**Total**	183(100%)	229(100%)			
**Working shift**					
**Day**	67(36.6%)	81(35.4%)	1.00		
**Night**	73(39.9%)	74(32.3%)	1.2(0.76–1.9)	0.21	1.45(0.81–2.59)
**Alternative**	43(23.5%)	74(32.3%)	0.70(0.43–1.2)	0.92	0.97(0.50–1.86)
**Total**	183(100%)	229(100%)			
**Intent to leave job**					
**Yes**	84(45.9%)	117(51.3%)	0.81(0.55–1.2)	0.02	0.48(0.26–0.88)*
**No**	99(54.1%)	111(48.7%)	1.00		
**Total**	183(100%)	228(100%)			
**Working unit**					
**Medical ward**	29(15.85%)	46(20.1%)	0.56(0.25–1.27)	0.13	0.43(0.15–1.29)
**Surgical ward**	55(30.1%)	65(28.4%)	0.75(0.35–1.61)	0.30	0.59(0.22–1.59)
**Pediatric ward**	29(15.85%)	37(16.2%)	0.70(0.3–1.6)	0.21	0.49(0.16–1.48)
**Emergency room**	32(17.5%)	45(19.7%)	0.63(0.28–1.4)	0.09	0.38(0.12–1.16)
**Intensive care unit**	20(10.93%)	20(8.7%)	0.89(0.36–2.2)	0.83	1.14(0.33–3.97)
**Gynecology/obs**	18(9.84%)	16(7%)	1.00		
**Total**	183100(%)	229(100%)			
**Resource adequacy**					
**Yes**	6(3.28%)	38(16.67%)	1.00		
**No**	177(96.72%)	191(83.8%)	5.7(2.4–14)	0.39	1.64(0.53–5.06)
**Total**	183(100%)	228(100%)			
**Work satisfaction**					
**Poor**	41(22.4%)	55(24%)	1.7(0.9–3.1)	0.52	0.74(0.29–1.86)
**Fair**	116(63.4%)	116(50.7%)	2.2(1.3–3.79)	0.40	0.70(0.31–1.60)
**Good**	26(14.2%)	58(25.3%)	1.00		
**Total**	183(100%)	229(100%)			
**Clear communication between nurses**					
**Yes**	70(38.3%)	117(51.1%)	1.00		
**No**	113(61.7%)	112(48.9%)	1.79(1.14–2.5)	0.70	0.90(0.51–1.56)
**Total**	183(100%)	229(100%)			
**QOL Perception**					
**Poor**	36(19.67%)	51(22.3%)	1.45(0.81–2.6)	0.47	0.73(0.31–1.71)
**Fair**	111(60.67%)	104(45.4%)	2.2(1.36–3.55)	0.98	0.99(0.47–2.08)
**Good**	36(19.67%)	74(32.3%)	1.00		
**Total**	183(100%)	229(100%)			

***Significant at P-value less than 0.001

**Significant at P-value less than 0.01

* Significant at P-value less than 0.05, COR = Crude Odd Ratio, AOR = Adjusted Odd Ratio, CI = Confidence Interval, 1 = Reference.

## Discussion

The magnitude of burnout among 412 study participant who were working in public hospitals of Harari regional state and Dire Dawa administration was 44.4%. This result is consistent with the study done in Amhara regional state among 358 nurses (42.5%) who participated in the study and also this study was also somewhat lower than the study which was conducted in the same area, Amhara regional state among 369 nurses which was 50.4%. The differences may be due to setting or difference in the geographical setting [16, 22].

Similarly the study conducted in Brazil, Minas Geris showed the magnitude of burnout among 116 nurses participated in the study was 47% which is consistent with this study. Also the study done in the same area (Reo de Janeiro) among 130 nurses was 55.3% [23, 24].

Additionally a multi-country, cross-sectional study conducted in 10 European countries involving 23,159 nurses working in surgical and medical wards reported burnout among nurses in different countries: England (42%), Finland (22%), Belgium 25%, Germany (30%), Poland (40%), Ire-land (41%), Norway (24%), Spain (29%), Netherlands (10%), and Switzerland (15%) [25]. When it was compared with this study, the magnitude of burnout among nurses in England, Poland and Ire-land was consistent with this study but the magnitude of burnout of this study was higher than the other 7 European countries. This may due to difference in sample size and difference in payment.

The study conducted in South west Ethiopia among 282 nurses and the study conducted in Mexico among 185 nurses revealed the magnitude of burnout which was lower than this study, which were 34% and 34.6% respectively [26, 27]. The discrepancy may due to the difference in sample size, difference in payment and the time in which the study was conducted. But the study done in US (united State) among 1000 selected nurses showed that the magnitude of burnout among 1000 selected nurses was 54.1%, which was higher than the study conducted on 412 nurses who were working in Harari regional state and Dire Dawa administration, Ethiopia [10]. This difference was may be due to difference in work load, difference in sample size and the time in which the study was conducted.

The nurses who were married were 2.3 times more risky for burnout than nurses who were single (p-value = 0.013). This association was not consistent with the study conducted in Amhara regional state, which showed reduced risk of burnout and married marital status [16]. The difference may due to income levels and other personal characters. Nurse who perceived their current health status as poor were 5 times more risk for burnout than nurses who perceived their current health status as good and also those nurses who perceived their current health status as fair have strong association with magnitude of burnout. Nurses with fair health status were 13.7 times more risky for burnout than nurses who perceives their health status as good (p-value<0.001). This finding is consistent with the study done in Amhara regional state which revealed increased risk of burnout by perceiving current health status as fair [16].

On the other hand, nurses who had an intention to leave their profession within a 12 months were also less likely to develop burnout than nurses who had no intention to leave their profession (AOR = 0.48 CI (0.28–0.88) with P-value = 0.02. This finding was consistent with the study conducted in Amhara regional state, Ethiopia [16]. Moreover the finding of this study also showed that, Nurses who were using anxiolytics, analgesic, smoking and who were doing physical activity in relation with their work related health problems were less risky in developing burnout.

## Strength and limitation of the study

### Strength of the study

This study had established some important point which will help us to generate hypothesis. It had showed the magnitude of burnout among nurses and used to see the relationship between the factors and burnout.

### Limitation of the study

Since the study was cross sectional, it couldn’t establish the cause and effect relationships.

### Conclusions

The magnitude of burnout among nurses who were working in public hospitals Harari regional state and Dire Dawa administration is high when compared to many other studies. Married nurses; perceiving current health status as poor and fair; having the intention to leave their profession within 12 months; working in emergency room; using anxiolytics and analgesics; smoking and physical activity were showed association with burnout among nurses. So, the concerned bodies should provide trainings which focus on stress copying mechanisms and assertiveness program and also give the attention and act on nurses’ burnout and factors which were associated with burnout among nurses. We also recommend the future researchers in order to conduct comparative and longitudinal study to verify the causal relationship between correlates and suicidal ideation and attempt.

## Supporting information

S1 File (SAV)
